# A cross-sectional study of *enteric fever* among febrile patients at Ambo hospital: prevalence, risk factors, comparison of Widal test and stool culture and antimicrobials susceptibility pattern of isolates

**DOI:** 10.1186/s12879-019-3917-3

**Published:** 2019-03-27

**Authors:** Tolera Deksissa, Endrias Zewdu Gebremedhin

**Affiliations:** 1 Department of Laboratories, West Shewa Health Bureau, Ambo Hospital, P.O. Box 03, Ambo, Ethiopia; 2 Department of Veterinary Laboratory Technology, Ambo University, College of Agriculture and Veterinary Sciences, P.O. Box 19, Ambo, Ethiopia

**Keywords:** Enteric fever, Widal test, Culture, Antimicrobial susceptibility, Risk factors and prevalence

## Abstract

**Background:**

Enteric fever is one of the common infectious diseases of humans. The objectives of this study were to:1) estimate the prevalence of enteric fever among febrile patients visiting Ambo hospital; 2) comparison of Widal test and stool culture;3) evaluation of the antimicrobial susceptibility of isolates; and 4) assess potential risk factors to acquire enteric fever infection.

**Methods:**

Blood and stool samples were collected from 372 febrile patients with symptoms clinically similar to enteric fever. Widal test was used for testing sera while stool culturing and bacterial identification was done using WHO standard methods. Susceptibility testing was done using Kirby-Bauer disc diffusion method. Chi-Square test and Logistic Regression analysis were used to analyze the data.

**Results:**

The apparent and true prevalence of enteric fever were 56.2% (95% confidence interval [CI]: 50.97–61.29%) and 57.52% (95% CI: 52.3–62.6%) respectively, while, the culture prevalence was 2.7% (95% CI: 1.30–4.89%). Isolation rates of *S*. Typhi and *S*. Paratyphi were 0.8% (95% CI: 0.17–2.34%) and 1.9% (95% CI: 0.76–3.84%) respectively. The isolates showed 100% resistance to amoxicillin, bacitracin, erythromycin, 80%resistance to cefotaxime and streptomycin and 20% for chloramphenicol. The sensitivity, specificity, positive and negative predictive values of Widal test was 80.0, 44.5, 3.8 and 98.8% respectively. Multivariable logistic regression analysis revealed that age (adjusted odds ratio [aOR] = 2.45; 95% CI: 1.38–4.37; *P* = 0.002), religion (aOR = 15.57, 95% CI: 3.01–80.64; *P* = 0.001), level of education (aOR = 2.60, 95% CI: 1.27–5.28; *P* = 0.009), source of water (aOR = 2.20, 95% CI: 1.21–3.98; *P* = 0.009), raw milk (aOR =2.19, 95% CI:1.16–4.16; *P* = 0.016) and raw meat consumption (aOR = 1.80, 95% CI: 1.07–3.01; *P* = 0.026) are the predictors of enteric fever seropositivity.

**Conclusions:**

Patients were wrongly diagnosed and treated for enteric fever by Widal test. Therefore, rapid tests with better sensitivity and specificity are needed for the diagnosis of enteric fever. Provision of safe water and health education are vital to bring behavioral change towards raw food consumption.

**Electronic supplementary material:**

The online version of this article (10.1186/s12879-019-3917-3) contains supplementary material, which is available to authorized users.

## Background

Typhoid fever is an acute systemic febrile illness caused by the bacterium *Salmonella enterica* serovar Typhi. *Salmonella enterica* serovars Paratyphi A, B, and C cause the clinically similar condition, paratyphoid fever [1, 2]. Typhoid and paratyphoid fevers are collectively known as enteric fevers [1]. While both diseases share clinical features, paratyphoid fever tends to have a more benign course of illness [3]. Enteric fever is an endemic disease in the tropics and sub-tropics primarily affecting children and young adults whereas in high-income countries it is mainly a disease of returning travelers [4]. The disease is most commonly acquired by ingestion of water and food contaminated with feces or urine of carriers. Human beings are the only reservoir host for enteric fever [1]. The illness due to enteric fever may be mild or severe but sometimes fatal. Enteric fever commonly presents with nonspecific clinical features such as fever, flu-like symptoms with chills, a dull frontal headache, malaise, anorexia, poorly localized abdominal discomfort, a dry cough and myalgia, nausea, vomiting, constipation, and diarrhea [1, 5, 6] which are indistinguishable from other causes of fever such as malaria.

Estimates of global burden of typhoid and paratyphoid fever indicated that in 2000, there were 22 million new cases of typhoid fever, 210,000 typhoid fever-related deaths, and 5.4 million cases of paratyphoid fever [7]. Developing nations share the highest burden due to rapid population growth, increased urbanization and limited safe water and health system [8]. The incidence of this neglected illness in some parts of South Asia is as high 1600 per 100,000 populations [9]. The case-fatality rate is higher in children under 5 years than school aged children and adolescents. It is highest in Sub-Saharan Africa and North Africa/Middle East regions [6]. In Ethiopia, enteric fever is among diseases claiming the life of many people particularly children. A study made in Ethio-Sewdish hospital in Addis Ababa (1984–1995) reported intestinal perforation in 27 patients (25%) of which 10 (37%) children died [5]. In untreated cases, the case fatality ranges between 10 and 30%, however, with appropriate and timely antimicrobial treatment it falls to 1–4% [7].

Isolation of *Salmonella* Typhi from blood culture is a more practical even though less sensitive alternative to bone marrow culture. However, it is not always available and, when available, it takes 3 to 5 days. As a result, diagnosis may be delayed or overlooked and patients without enteric fever may receive unnecessary and inappropriate antimicrobial treatment due to the heavy dependence of rapid diagnosis using clinical features and serological methods. The detection of specific antibody response is only suggestive of enteric fever but not definitive [10]. The Widal test is an agglutination reaction demonstrating the presence of lipopolysaccharide (LPS) somatic (O) and flagella (H) agglutinins to *Salmonella* Typhi in the serum of a patient using suspensions of O and H antigens. The test has been widely used for over a century for the diagnosis of typhoid fever [11].

In Ethiopia, like many other developing countries, diagnosis of enteric fever is usually made based of clinical evidence and results of Widal test. The Widal test is relatively cheaper, easy to perform and requires minimal training and equipment. Although the Widal slide agglutination test alone, mostly without tube dilution method, is routinely performed to detect presence of antibody production against enteric fever [12], the value of this test has been debated. Hence there is a need to evaluate the performance this test for correct interpretation of results. Culturing is not commonly done due to limited bacteriological facilities, trained professional and longer time required [11–13].

The emergence of antimicrobial resistance, especially the multidrug resistance (MDR) to ampicillin, chloramphenicol, and cotrimoxazole, is a major public health problem which further complicates the treatment and management of enteric fever [14]. Optimal antimicrobial treatment of patients with enteric fever depends on an understanding of local patterns of antimicrobial resistance and is enhanced by the results of antimicrobial susceptibility testing of the *Salmonella* isolated from the individual patient [14]. Because of the ready availability of over-the-counter antibiotics and subsequent resistance to these drugs in areas of endemicity, enteric fever is becoming harder to treat [9, 15].

Enteric fever is an endemic cause of febrile disease with major public health problems in Ethiopia. Studies on enteric fever have been undertaken in Ethiopia since 1970s. However, surveillance and monitoring systems for enteric fever are not in place [11, 16] and the numbers of studies published in literature don’t include all geographical regions and all segments of the population. Thus, comprehensive picture on the epidemiology of the disease such as on morbidity, mortality, risk factors and antimicrobial resistance is still inadequate [16]. Furthermore, the widespread existence of malnutrition, unhygienic living conditions and prevalence of HIV/AIDS are expected to exacerbate the situation. A recent meta-analysis and systematic review of salmonellosis in Ethiopia revealed that *Salmonella* Typhi and *Salmonella* Paratyphi were among the dominant serotypes isolated from diarrheal patients, febrile patients and apparently normal subjects [16]. In Ethiopia, it has been estimated that typhoid fever causes 3.3 annual mortality rate per 100,000 people. Typhoid fever, paratyphoid fever and other intestinal infectious diseases, respectively, have been reported as the three most deadly intestinal infectious diseases in Ethiopia during 2013 [17].

Therefore, the present study was initiated with the specific objectives to: 1) estimate the prevalence of typhoid/enteric fever; 2) comparison of Widal test and stool culture; 3) evaluation of the antimicrobial susceptibility of isolates; and 4) assess potential risk factors to acquire enteric fever infection among febrile patients visiting Ambo Hospital.

## Methods

### Study area and period

The study was conducted in Ambo town at Ambo hospital from February 2016 to December 2016. Ambo town is located in West Shewa Zone of Oromia Regional State, Ethiopia, 114 Km from Addis Ababa. Ambo town (latitude 8^0^59′N and longitude 37,051′E) has an elevation of 2100–2200 m above sea level. The mean annual temperature and annual rainfall of the area is 18 °C and 800–1200 mm respectively. The total population of Ambo town is 48,171 (24,634 males and 23,537 females. Ambo hospital gives service to patients coming from both urban and rural areas approaching 1357, 833 (677,599 males and 680,234 females) per year.

### Study design

The study was a health facility-based cross-sectional survey.

### Study population

Patients visiting Ambo hospital during the study period were the population for the study. Data from the hospital for the period from July 2014 to June 2015 indicated that out of the total patients attending the hospital, 6720 febrile cases were diagnosed as enteric fever. Presumptive febrile patients from outpatients’ departments (OPDs), wards (inpatients) and/or antiretroviral therapy (ART) clinic with symptoms clinically similar to enteric fever were the target population of the study.

### Sample size and sampling technique

The required sample size was calculated using the single proportion formula N = (Z) ^2^ P (1-P)/d2 [18] by considering expected prevalence (P) of 32.6% [8], 95% confidence interval (Z = 1.96) with a 5% margin of error or precision (d) and 10% non-response. Accordingly, the total sample size required for the study was calculated as 372.The representative groups were selected by simple random sampling.

### Inclusion and exclusion criteria

Febrile patients who were willing to participate in the study by giving informed consent; those who did not take antibiotic treatment for the enteric fever or for any other bacterial infections, and those who had fever for 2 or more days before visiting Ambo hospital accompanied by other clinical symptoms of enteric fever were included in the study. Those febrile patients who had received antibiotic treatment within 2 weeks before coming to the hospital and those who were diagnosed for other known febrile illness were excluded from the study.

### Questionnaire survey

Participants of the study were interviewed face to face by trained laboratory technicians using pre-tested structured questionnaire aimed to gather information on potential risk factors such as agro-ecology, age, sex, level of education, hand washing practice after visiting bath room (yes/no), use of toilet (yes/no), source of drinking water (pipe, stream/river), habit of drinking raw milk (yes/no), habit of eating raw meat (yes/no), habit of eating raw vegetables (yes/no), family size, marital status (single, married, widowed, divorced), occupation (government employee, self-employee, house wife, student) history of HIV/AIDS sero-status (positive/negative), previous history of typhoid treatment (yes/no). Other risk factors like, malaria, diabetes, tuberculosis (TB), cancer and organ transplantation were included in the questionnaire.

### Sample collection

The blood and stool samples were collected from those patients sent to the laboratory in the morning session (9 AM to 12 AM) of patient examination. Venous blood sample (2–3 ml) was collected aseptically using 70% alcohol in to a sterile test tube and centrifuged at 2500 revolution per minute for 5 min to separate the serum. Fresh stool specimen provided by febrile patient was put into screw capped container, labeled and transported to Microbiology Laboratory of Ambo University for culture.

### Laboratory examination

#### Serological test

Widal slide agglutination test was done using *S.* Typhi O and H antigens according to the instructions of the manufacturer. The antigen suspension commercially available in 5 ml volume from SPINREACT Reagent Ltd. (Spain) was used. A direct slide agglutination technique was used in this study for qualitative determination of the agglutination ability of sera. In brief, the test was done by mixing one drop of serum with one drop each of O and H antigens separately on slide. After shaking the slide back and forth for 1 min, the mixture was observed for macroscopic agglutination. If there was agglutination within 1 min it was reported as reactive, otherwise, non- reactive.

#### Isolation and identification

Stool sample was inoculated into selenite F broth (Oxoid, UK) and incubated for 24 h at 37 °C followed by subculture on xylose-lysine-deoxychocolate agar (XLD) (Oxoid, UK) and MacConkey agar (Oxoid) at 37 °C for 24 h for isolation of *Salmonella*. The growth of isolates was detected by their characteristic appearance on XLD agar (red colonies with a black center) and on MacConkey agar (pale colonies) [19].Suspected colonies were identified using standard phenotypic microbiological identification and serotyping techniques performed using serotype specific antisera.

#### Antimicrobial susceptibility testing

For antimicrobial susceptibility testing, the isolate was uniformly seeded over the surface of Mueller-Hinton agar (Oxoid, UK). Kirby-Bauer disc diffusion method was used for testing [20]. The drugs tested were ciprofloxacin (5 μg), chloroamphenicol (30 μg), gentamicin (10 μg), amikacin (30 μg), cefotaxime (5 μg), amoxicillin (30 μg), ceftriaxone (30 μg), streptomycin (10 μg) and bacitracin (10 μg). Standard operation procedure was followed to ensure the reliability and validity of test results. All the antimicrobials used for the study were purchased from Oxoid Ltd. Bashing store, USA.

### Quality control

As a means of quality control, the questionnaire prepared to assess potential risk factors was pre-tested before the main study on febrile patients. The data was collected by a trained laboratory technicians and nurses. The quality of culture media was tested for sterility and performance. Sterility of culture media was checked by incubating overnight at 35–37 °C without specimen inoculation. Any physical change like cracks, excess moisture, color, dehydration, and contamination were assessed and expiration date also checked. As a positive control standard strains of *E. coli* ATCC 25922 and *S. aureus* ATCC 25923 obtained from Ethiopian Public Health Institute were used during culture and antimicrobial susceptibility testing.

### Data analysis

Data generated from questionnaire survey and laboratory investigation were entered into Excel spread sheet (Microsoft Corporation). Then coded data was analyzed using STATA version 11.0 for Windows (Stata Corp. College Station, TX, USA). The data were summarized using descriptive statistics. The apparent prevalence of enteric fever was calculated as the number of seropositive/culture positive patients divided by the total number of patients examined multiplied by 100. True prevalence (TP) of enteric fever exposure was calculated by adjusting the apparent prevalence for specificity and sensitivity of the test using the formula, TP = AP+(Sp-1)/Se + (SP-1), [21], where AP is apparent prevalence, Sp is specificity and Se is sensitivity of the Widal test used [22]. Confidence limits for the proportions were established by exact binomial test with 95% confidence intervals (CI). The variables assessed were categorical and variables with more than two categories were transformed into indicator (dummy) variables. To identify explanatory variables of enteric fever seropositivity, first, the Pearson’s Chi-square test was used followed by univariable and multivariable logistic regression analyses. All non-collinear variables with *P*-value ≤0.25 in univariable analysis were considered for the multivariable logistic regression model to construct the likely model (*P* < 0.05). Sensitivity, specificity, positive predictive value (PPV), and negative predictive value (NPV) at 95% CI of Widal test was calculated by considering culture as gold standard. All statistical tests were considered significant if the *P*-value is ≤0.05.

## Results

### Socio- demographic data and results of serological test

A total of 372 febrile enteric fever suspected patients were enrolled during the study period. Majority (59.1%, 220/372) of the participants were females and the rest males (40.9%, 152/372). The age of the participants ranged from 7 to 82 years with mean + standard deviation (SD) of 31.4 + 13.4 years. Over 75.5% of the study participants were able to read and write and 10.7% had history of malaria medication. Overall, 209 patients were positive for enteric fever using Widal test giving an apparent or test prevalence of 52.6% (95% confidence interval [CI] = 50.97–61.29%). The true seroprevalence of enteric fever after correction for sensitivity (73.5%) and specificity (75.7%) of the testwas57.52% (95% CI:52.3–62.6%).The reactions of patients’ sera to O and H antigens using Widal test was presented in Table 1. Chi-square test showed that seropositivity to Widal test was significantly different with respect to age, sex, religion, source of water and meat consumption. The results of Widal test and its association with socio-demographic factors is presented in Table 2.

**Table 1:** Qualitative slide Widal agglutination test results of febrile patients suspected of typhoid fever in Ambo hospital, from February 2016 to December, 2016

Widal slide agglutination results	Number tested	% prevalence (95% CI)
Both O and H antigens reactive	184	49.5 (44.27–54.66)
Only O antigen reactive	25	6.7 (4.40–9.76)
Non-reactive for O and H antigens	163	43.8 (38.71–49.03)
Total	372	100

**Table 2:** Results of Widal test and its association with socio-demographic and other risk factors in febrile patients attending Ambo hospital, from February to December, 2016, (*n* = 372)

Variable	Category	No. tested	No. positive	% prevalence	X^2^	*P*- value
Agro-ecology	Highland > 2300 m	89	47	52.81	0.8376	0.658
	Midland 1900-2300 m	269	153	56.88		
	Low land < 1900 m	14	9	64.29		
Age	≥36 years	118	52	44.07	12.19	0.002
	7-18 years	49	26	53.06		
	19–35 years	205	131	63.90		
Sex	Male	152	74	48.68	5.87	0.015
	Female	220	135	61.36		
Religion	Protestant	185	90	48.65	12.53	0.002
	Orthodox	171	105	61.40		
	Others	16	14	87.50		
Education	Illiterate	91	47	51.65	1.96	0.581
	High School	70	38	54.29		
	Tertiary	74	41	55.41		
	Elementary	137	83	60.58		
Maternal status	Married	264	143	54.17	1.50	0.220
	Single	108	66	61.11		
Family size	6–8	110	59	53.64	1.53	0.675
	3–5	122	66	54.10		
	9–12	25	14	56.0		
	≤ 2	115	70	60.87		
Occupation	Gov’t employee	60	28	46.67	3.95	0.266
	Self-employee	95	51	53.68		
	Student	90	52	57.78		
	House wife	127	78	61.42		
Knowledge	No	249	135	54.22	1.18	0.277
	Yes	123	74	60.16		
Raw milk consumption	No	304	164	53.95	3.38	0.066
	Yes	68	45	66.18		
Raw meat consumption	No	119	55	46.22	7.06	0.008
	Yes	253	154	60.87		
Raw veg. consumption	No	233	130	55.79	0.038	0.845
	Yes	139	79	56.83		
Water Source	Pipe	257	135	52.53	4.51	0.034
	Stream	115	74	64.35		
Toilet in home	Yes	299	164	54.85	1.10	0.294
	No	73	45	61.64		
Other disease	Yes	62	30	48.39	1.83	0.175
	No	310	179	57.74		
Anti-acid	Yes	74	39	52.70	0.45	0.500
	No	298	170	57.05		
Hand wash	Yes	196	110	56.12	0.0006	0.98
	No	176	99	56.26		
Culture	Negative	362	201	55.52	2.37	0.124
	Positive	10	8	80.00		

Other = Daily Labor, Merchant, Farmer, pensioner, Driver, unemployed

#### Stool culture

Out of 372 febrile patients screened, stool culture revealed isolation of three *Salmonella* Typhi (0.8% prevalence, 95% CI = 0.17–2.34%) and seven *Salmonella* Paratyphi (1.9% prevalence, 95% CI = 0.76–3.84%) giving 2.7% (95% CI: 1.30–4.89%) culture prevalence of enteric fever. The sensitivity, specificity, positive and negative predictive values of Widal test as compared to the stool culture were 80.0, 44.5, 3.8 and 98.8% respectively.

#### Antimicrobial susceptibility pattern of *Salmonella* isolates

The isolates varied in their susceptibility to the antimicrobials used. Both *Salmonella* Typhi and *Salmonella* Paratyphi isolates were 100% resistant to amoxicillin, erythromycin and bacitracin. *S*. Typhi was 100 and 33.3% resistant for streptomycin and cefotaxime respectively. *Salmonella* Paratyphi was 100, 71.4, 28.6% resistant for cefotaxime, streptomycin, and chloramphenicol respectively. All isolates were 100% susceptible to ceftriaxone, gentamicin and amikacin (Table 3). Widespread multidrug resistance (MDR) i.e. resistance to more than two antimicrobial drugs, was detected in *Salmonella* isolates (Table 4).

**Table 3:** Overall antimicrobial resistance profile of *Salmonella* Typhi and *Salmonella* Paratyphi in Ambo hospital, from February 2016 to December, 2016

Antimicrobial drugs	Etiologic agents								
	*S*. Typhi			*S.* Paratyphi		Total			
	(N = 3)			(*N* = 7)		(*N* = 10)			
	S	I	R	S	I	R	S	I	R
AK	3	0	0	7	0	0	10	0	0
AML	0	0	3	0	0 0	7	0	0 0	10
B	0	0	3	0	0	7	0	0 0	10
CTX	2	0	1	0	0	7	2	0	8
CRO	3	0	0	7	0	0	10	0	0
C	1	2	0	5	0	2	6	2 2	2
CIP	2	0	1	7	0	0	9	0 0	1
E	0	0	3	0	0	7	0	0	10
CN	1	2	0	7	0	0	8	2	0
S	0	0	3	2	0	5	2	0	8

*AK* amikacin, *AML* amoxicillin, *B* bacitracin, *CTX* cefotaxime, *CRO* ceftriaxone, *C* chloramphenicol, *CIP* ciprofloxacin, *E* erythromycin, *CN* gentamycin, *S* streptomycin, *S* Susceptible, *I* Intermediate, *R* resistance

**Table 4:** Multidrug resistance (MDR) pattern of *Salmonella*Typhi and *Salmonella* Paratyphi in Ambo hospital, from February 2016 to December, 2016

MDR pattern	Resistance Pattern	No. of Isolates	% MDR
Two	AML, B	9	90
Three	AML, B, CTX	8	80
	AML, B, E	4	40
Four	AML, B, CTX, E,	4	40
Five	AML, C, CIP, CTX, E	1	10
	AML, B, CTX, E, S	1	10
Six	AML, B, C, CTX, E, S	1	10

#### Associated risk factors

Univariable logistic regression analysis showed significant association between seroprevalence of enteric fever and age (*P* = 0.001), sex (*P* = 0.016), religion (*P* = 0.016, *P* = 0.009), consumption of raw meat (*P* = 0.008) and water source (*P* = 0.034) (Table 5).

**Table 5:** Results of logistic regression analysis of potential risk factors and their association with seroprevalence of typhoid fever among febrile patients in Ambo hospital

		Univariable		Multivariable	
Variables	Categories	OR (95% CI)	*P*-value	OR (95% CI)	*p*-value
Age	≥36 years	1.00	–	1.00	–
	7–18 years	1.43 (0.74, 2.80)	0.290	1.19 (0.42, 3.39)	0.743
	19–35 years	2.25 (1.42, 3.57)	0.001	2.45 (1.38, 4.37)	**0.**002
Sex	Male	1.00	–	1.00	–
	Female	1.67 (1.10, 2.54)	0.016	1.51 (0.83, 2.73)	0.174
Religion	Protestant	1.00	–	1.00	–
	Orthodox	1.68 (1.10, 2.56)	0.016	2.12 (1.31, 3.45)	0.002
	Others	7.39 (1.63, 33.43)	0.009	15.57 (3.01, 80.64)	0.001
Education	Illiterate	1.00	–	1.00	–
	High school	1.11 (0.60, 2.56)	0.740	1.75 (0.76, 4.03)	0.187
	Tertiary	1.16 (0.63, 2.15)	0.631	2.66 (0.94, 7.54)	0.065
	Elementary	1.44 (0.84, 2.46)	0.183	2.60 (1.27, 5.28)	0.009
Marital status	Married	1.00	–	1.00	–
	Single	1.33 (0.84, 2.10)	0.221	2.78 (0.97, 7.98)	0.058
Family size	6–8	100	–	1.00	–
	3–5	1.02 (0.61, 1.71)	0.944		
	9–12	1.10 (0.46, 2.64)	0.831		
	≤2	1.34 (0.79, 2.28)	0.273		
Occupation	Gov.employee	1.00	–	1.00	–
	Self-employee	1.32 (0.69, 2.53)	0.395	1.59 (0.64, 3.97)	0.317
	Students	1.56 (0.81, 3.02)	0.182	1.12 (0.37, 3.71)	0.790
	House wife	1.82 (0.98, 3.38)	0.059	2.46 (0.94, 6.43)	0.067
Knowledge	No	1.00	–		
	Yes	1.28 (0.82,1.98	0.277		
Raw milk	No	1.00	–	1.00	–
consumption	Yes	1.67 (0.96, 2.90)	0.068	2.19 (1.16, 4.16)	0.016
Raw meat	No	1.00	–	1.00	–
consumption	Yes	1.81(1.17, 2.81)	0.008	1.80 (1.07, 3.01)	0.026
Raw veg.	No	1.00	–		
consumption	Yes	1.04 (0.68, 1.59)	0.845		
Water source	Pipe	1.00	–		
	Stream	1.63 (1.04, 2.57)	0.034	2.20 (1.21, 3.98)	0.009
Toilet in home	Yes	1.00	–		
	No	1.32 (0.78, 2.23)	0.295		
Other disease	Yes	1.00	–	1.00	–
	No	1.46 (0.84, 2.52)	0.177	1.48 (0.78, 2.82)	0.229
Antacid use	Yes	1.00	–		
	No	1.19 (0.72, 1.99)	0.501		
Hand washing	Yes	1.00	–		
	No	1.01 (0.67, 1.51)	0.980		
Culture	Negative	1.00	–	1.00	1.00
(S.T, *S.*P)	Positive	3.20(0.67, 15.30)	0.144	3.72 (0.71,19.58)	0.121

*OR* odds ratio, *CI* confidence interval, other = daily labor, pensioner, merchant, farmer and driver. The bold *P*-values indicate significant association

Other religion = Muslim, Wakefata, Kalu, Abagada

*S.* T *Salmonella* Typhi, *S.* P *Salmonella* Paratyphi

Multivariable logistic regression analysis revealed that age, religion, level of education, raw milk consumption, raw meat consumption and source of water are independent predictors of Widal test seropositivity (*P* ≤ 0.05). The Odds of enteric fever seropositivity for age group 19–35 years was 2.45 times higher compared to those ≥36 years of age (95% CI of OR = 1.38–4.37, *P* = 0.002). Similarly, the risk of enteric infection was 2.12 times higher in Orthodox than Protestant (95% CI: 1.31–3.45, *P* = 0.002) religion followers. The risk of enteric infection was 2.6 times higher in those who had elementary education (95% CI: 1.27–5.28, *P* = 0.009) than illiterate groups. Those respondents who consumed raw milk are 2.19 times more likely to get infected with enteric fever than those who consumed boiled or pasteurized milk (95% CI: 1.16–4.16; *P* = 0.016). The likelihood of acquiring enteric fever was 1.8 times higher in those febrile patient who eat raw meat than those who didn’t consume raw meat (95% CI: 1.07–3.01; *P* = 0.026). The risk of enteric fever infection was 2.2 times higher in patients who drunk stream water when compared with those who drink pipe water (95% CI: 1.21–3.98; *P* = 0.009) (Table 5).

## Discussion

The present study identified high Widal seroprevalence, low prevalence from stool culture (*S.* Paratyphi being dominant isolate), widespread multidrug resistant *S*. Typhi and *S*. Paratyphi isolates and six important risk factors or independent predictors (age, religion, education, source of drinking water, raw milk and raw meat consumption habit) for acquiring enteric fever infection among febrile patients visiting Ambo hospital.

### Slide Widal agglutination test

Diagnosis of typhoid fever using Widal slide agglutination test was common in the current study area and subsequent treatment of patients relies on this test result due to the absence of culture facilities [23].The serological prevalence of enteric fever in this study was 56.2%, while only 10 (2.7%) febrile patients were culture proven to have enteric fever agents. The government of Ethiopia has achieved coverage of safe water to 54% of households, however, nearly 39 million people, most of them in rural areas, don’t have access to safe water and nearly 48 million lack access to basic sanitation [24]. Considering the above fact, the high seropositivity found in the current study is justifiable. Inadequate food and personal hygiene; and lack of potable water have been frequently cited as major risk factors contributing for high seropositivity of enteric fever [1].Moreover, people who live in enteric endemic areas like Ethiopia are supposed to have background antibody due to old infection or repeated infection with agents causing enteric fever or other *Salmonella* serotypes, that can react with Widal test. Therefore, a single Widal test might not be of great diagnostic relevance in adolescents and adults residing in endemic areas as it leads to over diagnosis and over-reporting. The background or baseline level of antibody to enteric fever determined in healthy population in Ethiopia showed that almost all the blood tested showed some titer of the antibody reactive to slide agglutination tests [25]. This shows low clinical diagnostic reliability of Widal test [12, 23].False positivity in Widal test could also exist due to infection with other *Enterobacteriacae* possessing cross-reacting epitopes and cross reaction with malaria [1, 26].False negative Widal test result might arose due to testing of patients at early stage of the disease (acute phase) before full antibody response or prior antibiotic treatment or ingested bacterial load is inadequate to induce the antibody production. Such negative Widal test result makes exclusion of enteric fever difficult in a patient with history and signs matching with enteric fever. Thus, the inadequate sensitivity and low specificity of Widal test kits coupled with treatment of patients using single Widal test result alone might have resulted in mistreatment or unnecessary treatment or undesirable treatment outcome or missed appropriate treatment opportunities [12, 23, 26].

The present study showed that the serological prevalence of enteric fever (56.2%) is high while culture prevalence (2.7%) is relatively low. Similar results have been reported previously from Khartoum [13], Nigeria [27] and Ethiopia [23]. As opposed to our study, low seroprevalence of typhoid fever using Widal test, i.e. 19% (38/200) [26] and14.1% [12] have been reported in febrile patients at a Rural Health Center in Northwest Ethiopia and in a 5-year (2007–2011) retrospective study in Addis Ababa, respectively. Similarly, Animut et al. [28] reported low seroprevalence of enteric fever (5.8%) in febrile patients of Quarit, Dembecha and Jabitehnan districts of Northwest Ethiopia.

The sensitivity, specificity, positive and negative predictive values of Widal test as compared to the gold standard test (culture) were 80.0, 44.5, 3.8 and 98.8% respectively. This show that the Widal slide agglutination test had low specificity and low positive predictive values but high sensitivity and negative predictive values. A negative Widal test result is a good indicator of the absence of enteric fever. Similarly, a study conducted in Ethiopia indicated that a negative result of Widal test would have a good predictive value of the absence of the disease (NPV = 98.9%) while a positive result would have a very low value for enteric fever (PPV = 5.7%) [8].

### Stool culture

The overall culture prevalence of *Salmonella* Typhi and *Salmonella* Paratyphi isolates in this study was 2.7% suggesting that these positive patients might be source of infection for healthy individuals through food handling and contamination of the environment. This low prevalence might be attributed to irregular shedding of the bacteria [1] and to the fact that the majority of the patients might have come from urban areas with relatively better accesses clean water and health care settings. On the other hand, attribution of the isolation rate to clinical disease should be done cautiously since enteric fever is endemic in Ethiopia and carriers do exist. This is similar with the study conducted in Bahir Dar University among food handlers (2.7%) [29]. A relatively higher isolation rate of typhoidal *Salmonella* was reported from other regions of Ethiopia such as Addis Ababa (3.5%) [30], Arba Minch University (6.9%) [31] and Jimma (6.2%) [32]. However, compared to the present isolation rate, much lower percent of isolation (0.5%, 1/200) was reported through culturing of blood of febrile patients in Northwest Ethiopia [26].This varied prevalence might be due to difference in year of study, sampling technique used, laboratory methodologies used, ecological variations among study sites, difference in cultural and hygienic practices, volume of stool examined, time of sample collection (patients with a history of fever for 7–10 days being more likely than others to have positive culture) and the type of sample cultured (stool versus blood) [1, 19].

Considering the availability of drugs without prescription and the practice of initiation of treatment at home and health-seeking behavior of people when such treatments fail, it is difficult to trust the response of all respondents interviewed aimed at exclusion of those who started antimicrobial usage before coming to hospital. Thus, the low isolation rate might also be partly attributed to initiation of self-prescribed antimicrobial therapy before visiting the hospital.

The high rate of *S*. Paratyphi isolation (7/10) as compared to *S*. Typhi (3/10) in the present study, in the absence of *S*. Typhi vaccination programs in Ethiopia, worth mentioning. This might show *S*. Paratyphi is the dominant etiological agent of enteric fever circulating in the study area. However further large-scale studies are required to ascertain our finding before considering *S.* Paratyphi as emerging agent of enteric fever. In accord with our finding, recently, Mahapatra et al. [33] in India isolated 167 *Salmonella* Spp in a 5-yearretrospective study and reported emerging enteric fever caused by *S*. Paratyphi due to a dramatic increase in the isolation of *Salmonella* Paratyphi A (83.8%) than *S*. Typhi (16.6%) during 2008–2011.

The interesting findings of the present study are that out of the 10 culture positive cases 8 were Widal positive and two febrile patients culture positive for *S*. Typhi were Widal negative (data not shown). This shows that the humoral immune response confers incomplete protection against relapse or re-infection [1]. Similar to our finding Shlim et al. [34] and Tankhiwale et al. [35] reported that increasing proportions of enteric fever is due to paratyphoid fever. The high rate of isolation of *S*. Paratyphi than *S*. Typhi together with antimicrobial resistance of *S.* Paratyphi might suggest important health challenge in the absence of effective vaccine for paratyphoid fever [3].

### Antimicrobial resistance

In this study, *Salmonella* isolates showed high rate of resistance to a number of commonly used antimicrobial drugs in Ethiopia such as amoxicillin (100%), bacitracin (100%), erythromycin (100%), cefotaxime (80%), streptomycin (80%) and chloramphenicol (20%). This is in agreement with the findings of Aklilu et al. [30] who reported 100% resistant *Salmonella* isolates to amoxicillin and erythromycin. In our study, all enteric fever isolates were susceptible for ceftriaxone and gentamicin (100%), hence these drugs could be considered for treatment of enteric fever. All three *S*. Typhi isolate were susceptible for chloramphenicol, 28.6% of *S*. Paratyphi isolates were resistant for chloramphenicol while 33.3% of *S.* Typhi and 100% of *S*. Paratyphi isolates were resistant to cefotaxime. The relatively lower resistance (20%) to the conventional antibiotic, chloramphenicol, that has been used for so long is not to our expectation. Similarly, Mahapatra et al. [33] from India reported low level of resistance to chloramphenicol. Contrary to our study, high resistance rate of typhoid agents to ceftriaxone (75%), chloramphenicol (83.7%) and gentamicin (75.6%) have been reported in other part of Ethiopia [36]. The detection of one *S*. Typhi isolate (1/10, 10%) resistant for ciprofloxacin is of concern because this antibiotic is commonly used for treatment of other bacterial infections. It is also used to treat enteric fever due to emergence of MDR to first line drugs like chloramphenicol, ampicillin and cotrimoxazole.

The high MDR among enteric fever isolates suggests that patients treated with those antimicrobial drugs (like amoxicillin) might have had high frequency of poor clinical response, severe disease with potential complications and adverse outcomes [1, 6] as compared to drug sensitive enteric fever isolates. The high rate of MDR also leads to limited therapeutic options and high financial implications. Thus, rational use of antimicrobial drugs and occasional antimicrobial drug resistance surveillance and monitoring are essential to limit the further evolution of multi-drug resistance [6].

Factors which contribute for emergence of AMR are often complex involving the interplay of human, environmental and pathogen-related factors [37, 38].Drug resistance to etiological agents of enteric fever in the present study might probably be due to over- prescription, indiscriminate prescription [23] ease of access (cheap, widely available) to antibiotics [6, 36], antibiotic use practices including self-medication, high frequency of antibiotic use, sub-therapeutic use or indiscriminate use [15] and substandard antibiotic preparations [6].It might also be due to the transfer of resistance genes (plasmid mediated) between enteric bacteria or through chromosomal mutation, that make the bacteria, impermeable to the drugs. In recent decades emergence of plasmid-encoded MDR, particularly to the quinolones has been reported as a major problem [33].

### Risk factors

The risk factors identified in the study area showed that seroprevalance is significantly associated with age, religion, level of education, source of water, raw milk and raw meat consumption which might play a major role in the endemicity of typhoid fever in the area.

The high rate of urbanization in Ethiopia and its consequent increasing number of people living in slum where sanitary facilities and provision of clean water are not fulfilled coupled with the poor municipal waste management, might predispose people to use fecal contaminated stream water for food preparation and drinking purpose Discharge of inadequately treated sewage and run-off storm water into the environment can lead to deterioration of quality of water sources [39]. In many studies unsafe contaminated water sources have been linked to enteric fever [40, 41].

Human beings are the major reservoir for enteric fever causative organisms [1] thus milk from healthy cows is not expected to contain agents of enteric fever. However, inadequate hygiene of milkers’ who might be carriers, the use of contaminated water for washing hands, udder and teats and milking utensils might contribute for contamination of milk [18] and high seropositivity in patients who consumed raw milk as compared to those who consume pasteurized or boiled milk. In a review of 68 epidemiological investigations of typhoid fever outbreaks Glynn and Bradley [42] reported milk and ice cream to account for 11 (16%) and 3 (4%) of the outbreaks, respectively. Nevertheless, studies on epidemiology and environmental drivers of enteric fever infection in Africa is limited [43] for further comparison.

Consumption of raw meat, identified as risk factor in the present study, is a well-known and widespread traditional practice in Ethiopia. Carcasses from apparently healthy animals are generally assumed to be free of *Salmonella.* Contamination of carcass with *Salmonella* Typhi and *Salmonella* Paratyphi from food handlers, who might be chronic carriers [29], could occur at different points of the meat chain from abattoir until consumption. Inadequate carcass handling, and inadequate hygienic standards of the people working in the slaughter houses, butchers and restaurants have been previously reported as source of contamination in Ethiopia [29, 44].The use of the same knives and cutting boards for infected and uninfected meat in butcheries and restaurants contribute for cross contamination. The use of unsafe water for washing of carcasses and food contact surfaces might also expose carcass surface and susceptible fleshy parts for contamination [45]. Similar to our finding, Tassew et al. [46] also reported consumption of raw and inadequately cooked meat to be potential risk factors of enteric infection.

The risk of typhoid infection was high in the age group of 19–35 years which is in accord with a study conducted in another region of Ethiopia [12, 30, 31]. This might be due to demographically and economically active and productive individuals in the communities, who are more exposed to occupational hazards of farming related water contact activities, contaminated environment, food and drinks that might be eaten out doors and the nature of the environment in which the food and drink are prepared [47].

People from elementary education are at higher risk (adjusted OR = 2.60, 95% CI: 1.27–5.28; *P* = 0.009) of acquiring typhoid infection perhaps due to their low level of knowledge and awareness which predispose them to various sources of infections such as from contaminated soil, water, hands and food. In accord to our findings Sutiono et al. [41] from Indonesia reported education as significant risk factor for typhoid fever prevalence. Unlike our findings, Birhanie et al. [26] reported absence of significant association between typhoid fever and source of water as well as educational background. The same authors also reported significant association between hand washing habit and typhoid fever infection, which is different from our finding.

Febrile patients with a Religion categorized as others (Muslims, Abageda, Wakeyfeta) are more likely to acquire enteric fever (adjusted OR = 15.57, 95% CI: 3.01–80.64; *P* = 0.001) as compared to febrile patients of Protestant Religion. No logical explanation could be given for the significantly high seropositivity in Muslim and Orthodox as compared to Protestant Religion followers. It was difficult to give valid conclusion on this risk factors as it might be due to chance or the small sample size of other Religion followers in the present study. Therefore, further investigation is required to give a more valid conclusion on the role of Religion as risk of enteric fever seroprevalence. Nevertheless, Sur et al. [40] from India reported more frequent case of enteric fever among Muslim than Hindu communities..

Results of Widal seropositivity was significantly high in females (61.36%) than males (48.68%) using univariable logistic regression analysis (*p* = 0.016), however, sex was not independent predictor in the multivariable model. The household activities of women in food preparation, child and patient care might expose them more to typhoid infection than males [26].

One of the strength of our study is that we assessed about 17 putative factors for their association with enteric fever seropositivity using appropriate model. Findings from this model could be used for improving the control strategies. Nevertheless, it is worthy to consider our findings in light of some limitations such as failure to determine end titer of antibodies, failure to screen blood films to rule out malaria and other febrile illnesses, failure to culture from blood and failure to perform antimicrobial susceptibility testing using all recommended drugs due to shortage. Moreover, the findings are from hospital based cross-sectional study and interpretation of the results to the general population should be cautiously done. Rather our data provide a platform for population based studies in order to ascertain the burden of typhoid fever.

## Conclusions

The serological prevalence of enteric fever using Widal test was high while, culture prevalence was low, hence there is substantial discrepancy between Widal test and stool culture for diagnosis of enteric fever. Age, level of education, religion, source of water, raw meat and raw milk consumption are associated risk factors for enteric fever. The *Salmonella* Typhi and *Salmonella* Paratyphi isolates showed high MDR to the commonly used antimicrobial drugs in Ethiopia. Other tests which are highly specific and better than Widal test are urgently needed to diagnose enteric fever in Ethiopia as some patients have been wrongly diagnosed and treated for the disease using the Widal test. Besides, it is imperative to undertake health education, improvement of water supply and boiling of stream water before consumption, provision of sanitation facilities, periodic surveillance and antimicrobial susceptibility testing and appropriate use of antimicrobial drugs as a means to combat enteric fever. Further epidemiological studies in community settings rather than hospital-based sampling is essential to provide a more reliable information at population wide level in order to curtail the burden of enteric fever.

## Additional file

Additional file 1: Questionnaire. (DOC 32 kb)

## Abbreviations

AK: Amikacin
AML: Amoxicillin
AMR: Antimicrobial resistance
aOR: Adjusted odds ratio
Ap: Apparent prevalence
ART: Antiretroviral therapy
ATCC: American type culture collection
B: Bacitracin
C: Chloramphenicol
CI: Confidence interval
CIP: Ciprofloxacin
CN: Gentamycin
CRO: Ceftriaxone
CTX: Cefotaxime
E: Erythromycin
HIV/AIDS: Human immunodeficiency virus/acquired immunodeficiency syndrome
I: Intermediate
LPS: Lipopolysaccharide
MDR: Multidrug resistance
NPV: Negative predictive value
OPD: Outpatients’ departments
PPV: Positive predictive value
R: Resistance
S: Streptomycin
S: Susceptible
S. Paratyphi: Salmonella Para typhi
S. Typhi: Salmonella Typhi
Se: Sensitivity
Sp: Specificity
TP: True prevalence
USA: United states of America
WHO: World Health Organization
XLD: Xylose-lysine-deoxychocolate, μg Micro
