# Supplementary material for: A cross-sectional study of enteric fever among febrile patients at Ambo hospital: prevalence, risk factors, comparison of Widal test and stool culture and antimicrobials susceptibility pattern of isolates
Source: BMC Infect Dis. 2019 Mar 27;19:288. doi: 10.1186/s12879-019-3917-3 (PMC6437987; doi:10.1186/s12879-019-3917-3)
Supplement: Supplementary file 1 — Questionnaire. (DOC 32 kb) [file 12879_2019_3917_MOESM1_ESM.doc]

Questionnaires (English Version)

Participants were asked the following questions. These questions were used to collect information on potential risk factors to acquire enteric fever.

Date of interview:_____________card.No-_____________Laboratory Code_____________

Part one. Address of the patients

1. Region__________________________ 2. Zone ________________________

3. Woreda________________________4.Kebele_______________________

5. Card No_________________________6. Tel________________________

Part two. Socio demographic Information

7. What is your age in years?___________________

8. Sex? 1. Male 2. Female

9. Religion 1.Orthodox 2. Muslin 3.Protestant 4.Catholic 5. Other

10. Ethnicity? 1. Oromo 2. Amhara 3.Gurage 4.Tigre 5. Other, specify,___________________

11. Educational Level 1. Illiterate 2. Elementary 3. High School `

4. College 5. University

12. Marital Status 1. Single 2. Married 3. Separate 4. Divorced 5. Widowed

13. Number of family members ___________________

14. Occupation 1. Unemployed 2. Civil servant 3. Student 4. House wife 5. Daily laborer 6. Pensioned 7. Merchant 8. Farmer 9. Driver 10. Other

15. Monthly income on Birr______________

Part III. Current clinical presentation

15. Headache 1. Yes 2.No. if yes duration_______________

16. Fever 1.Yes 2. No If yes duration_______________

17. Abdominal pain 1.Yes 2. No If yes Duration_______________

18. Bloody diarrhea 1. Yes 2. No

19. Chill 1. Yes 2. No

20. Weakness 1. Yes 2. No

**Part Four**: Predisposition for Typhoid fever

21. Do you know /you have some information about typhoid? 1. Yes 2.No 3. I don’t know

22. Habit of drinking milk 1. Raw 2. Boiled 3. Both (boiled & raw) 4. I do not drink

23 Do you have the habit of eating raw meat 1. Yes 2. No

Do you have the habit of raw vegetable/salad/ consumption? Yes/no

24. Do you have toilet in your home 1. Yes 2. No If No, where are you use?___________________________________________________-

25. Do you have history of hospital admission 1. Yes 2. No

26 Do you know your HIV test result? 1. Yes 2. No

If yes, what was your HIV test result? 1. Positive 2. Negative

27. Are you pregnant (for Female) 1. Yes 2. No

28. Previous history of typhoid fever treatment_____________________

29. Do you have a habit of drinking pipe water 1. Yes 2. No If No, what type of water you use for drinking and preparation of food____________________________________________

31. Did you use Anti acid recently 1. Yes 2. No

32. Do you have the habit of washing your hands carefully with soap and water after using the bathroom 1.Yes 2. No

33. Do you prepare or serve food for other people? 1. Yes 2. No

34. Please indicate which of the following disease you have

a. Diabetes 1. Yes 2. No

b. Cancer 1. Yes 2. No

c. AIDS 1. Yes 2. No

d. Malaria 1. Yes 2. No

e. Transplantation 1. Yes 2. No

Other:specify___________________________________________________________________

Name and signature of the data collector/interviewer______________________________
